# Multiphase simulation of sustainable nanoenhanced ionic liquid coolants for improved thermal performance in Ti–6Al–4V alloy drilling

**DOI:** 10.1016/j.heliyon.2023.e23020

**Published:** 2023-11-29

**Authors:** B. Srivathsan, Thaniarasu G, K. Vishnu Ram, Harish R

**Affiliations:** School of Mechanical Engineering, Vellore Institute of Technology, Chennai, Tamil Nadu-600127, India

**Keywords:** Ionic liquid, Nanocoolant, Carbon nanotube, Drilling temperature, Titanium alloy

## Abstract

Extensive research has been conducted by the manufacturing industry to enhance the efficiency of drilling processes by focusing on the utilization of nanoenhanced cutting fluids that possess excellent heat conductivity. Due to their eco-friendliness and adaptability of physical and chemical properties, ionic fluids offer enormous potential for application as cutting fluids. This study investigates the computational fluid dynamics analysis of the heat transfer performance of various ionanofluid pairs dispersed with nanoparticles as cutting fluids in the drilling process using Ansys Fluent software. For this purpose, 1-Hexyl-3-methyl-imidazolium-tetrafluoroborate is considered the ionic fluid, and its thermal behavior is examined by dispersing it with nanoparticles of copper, silver, and multiwalled carbon nanotubes (MWCNT) at different particle volume fractions and Reynolds numbers. The workpiece is composed of an alloy of titanium Ti–6Al–4V, while the drill bit is made of tungsten carbide-cobalt. It is observed that the ionic nanocoolant mist emanates from the spray tip and moves towards the drill bit-workpiece interface. Initially, the coolant's velocity is greatest close to the orifice, and as time passes, it approaches the drilling space. The data indicates that the spraying velocity of the coolant augments over time and that it disperses heat at the tool-chip interface. The results help us validate the flow and interaction of ionanocoolant with the drilling zone. With a rise in the volume fraction of added nanoparticles and Reynolds number, the results indicated a significant decrease in the drilling temperature. With a higher particle volume fraction, the MWCNT-ionic coolant combination decreases the drilling temperature of pure ionic liquid by 25.64 %. The copper, silver, and MWCNT ionanofluids enhance the average heat transfer coefficient of pure ionic coolant by 35.14 %, 47.42 %, and 62.75 %, respectively. In addition, MWCNT nanocoolants demonstrated improved thermal performance and heat removal rate in comparison to copper and silver ionanocoolants.

## 1 Introduction

In the realm of the manufacturing industry, addressing heat dissipation challenges during machining processes is crucial for optimizing performance and efficiency. As machining technologies advance, the demand for efficient cooling and lubrication methods has grown exponentially. Nanoparticle-enhanced ionic liquid machining coolants have emerged as a ground-breaking solution in this context. By combining the exceptional heat transfer properties of nanoparticles with the unique attributes of ionic liquids, these innovative coolants have revolutionized the way heat is managed during machining operations. The benefits of such coolant systems are manifold. They not only maximize heat extraction but also minimize thermal damage, facilitating increased machining speeds, extended tool life, and superior surface finish. Sultana and Dhar [1] conducted a computational investigation into the machining process of Ti–6Al–4V alloy by employing high-pressure coolant jets. Their computational investigation highlighted the forced convection heat transfer phenomena associated with this approach, revealing a notable reduction in cutting force and friction due to controlled heat generation. However, the importance of effective coolant systems becomes even more evident when we consider the risks of inadequate lubrication. Girinon et al. [2] examined the potential hazards associated with inadequate lubrication in drilling processes. While the circulation of coolant effectively managed heat dissipation, the absence of it in dry machining led to heightened thermal exposure of the workpiece, making it susceptible to crack formation. From an economic perspective, the implications of coolant and tooling are not to be overlooked. In a standard production process, the expenses related to cutting fluids and tools constitute a significant chunk of the overall manufacturing cost, estimated at up to 17 % and 4 %, respectively. Di et al. [3] observed that high temperatures generated at the tool-chip surface during milling operations resulted in several adverse effects, including rapid tool wear and a decrease in the strength and effectiveness of the cutting tools, which increased the necessity of coolant in machining processes. The quantity of heat that may be generated during the machining process has a substantial effect on the final product's quality and the amount of energy consumed. According to Baohai et al. [4] milling of hard materials leads to excessively high contact temperatures between the tool and chip. Reduced heat conductivity in the employed coolant causes considerable friction. This diminishes tool durability, tool quality, and productivity. In recent machining applications, the usage of novel cutting tool materials and cutting fluids has increased, which can alter the rate at which materials are removed from the workpiece and the quality of the final result. Metalworking fluids, also known as cutting fluids, are employed as a coolant in the machining process to diminish the heat production attributed to the frictional forces between the cutting tool and the workpiece interface. According to Aparicio et al. [5], in recent years, both industry and academics have shown a heightened interest in ionic liquids as a result of the expanding demand for environmentally acceptable fluids to meet national and international environmental standards. According to Qu et al. [6], room-temperature ionic liquids are predominantly used as "green" solvents in chemical synthesis and electrochemistry because of their exceptionally low vapour pressure and good thermal stability. Gupta et al. [7], studies have concentrated on the thermal properties of these solid particles dispersed in ordinary fluids because solid particles exhibit higher thermal conductivities. These micrometer-sized nanoparticles could be added to the base fluid to improve its ability to transmit heat [8]. Since Choi and his colleagues discovered nanofluids in 1995, there has been a rise in the amount of academic research on nanocoolants [9]. Visconti et al. [10] established a model that compared the utilization of nanofluids as a heat transfer fluid with a traditional model. Their findings revealed that employing nanofluids not only enhances efficiency but also leads to cost reductions.

In their study, Chakraborty and Panigrahi [11] provide a comprehensive analysis of various approaches to nanofluid stability in diverse applications, such as heat transfer, microfluidics, and lubrication. Ghadimi et al. [12] analyzed the characterization of nanofluids, focusing on thermal conductivity and viscosity while investigating theories such as Brownian motion and particle aggregation. Abdulhameed et al. [13] conducted simulations using the finite element method to examine the effects of dielectrophoresis and AC electroosmosis on conductive and insulating particles. Their findings revealed distinct responses from carbon nanotubes and polystyrene particles to different frequencies, providing a method for future multilayer fabrication. A similar study also explored a new dielectrophoresis setup for assembling carbon nanotubes on electrodes, effectively reducing drag velocity for microfluidics applications [14]. Among various nanoparticles, carbon nanotubes [15,16] are distinguished by their exceptional thermal performance, effectively enhancing the rate of heat transfer.

A potential option for heat transfer fluids in machining applications is nanofluids, which have been discussed in studies on nanocoolants [[17], [18], [19], [20], [21], [22]] and are explored further in the context of nanofluids and minimum quantity lubrication [[23], [24], [25], [26]]. The role of carbon nanotubes in improving thermal and electrical conductivity has been widely studied [[27], [28], [29]].

The incorporation of Al_2_O_3_ nanoparticles enhances the drilling characteristics in terms of drill tip temperature, hence enhancing the heat removal and lubricating qualities of coolants. In addition, nanofluid minimum quantity lubrication lessens the severity of tool adhesion in comparison to dry and flood circumstances [30]. Nanocoolants have improved the ability to diffuse heat compared to oil and water. Particle aggregation and sedimentation decrease heat performance and increase viscosity. Das et al. [31] compared the heat removal rates using various nanocoolants, which were prepared by mixing nanoparticles of ZnO, CuO, Fe_2_O_3_, and Al_2_O_3_ in deionized water, during the turning operation of AISI 4340 steel. The findings indicated that the surface finish of the tool significantly improved when the nanofluid contained CuO nanoparticles compared to the other three combinations. CuO-based nanofluid has a lower viscosity than ZnO, Al_2_O_3_, and Fe_2_O_3_ based nanofluids, which explains the enhanced surface polish. This also led the nanofluid to settle appropriately at the interface between the workpiece and the tool. Singh et al. [32] evaluated the thermal performance of various nanocoolants in grinding operation. Jerold et al. [33] utilised cryogenic CO_2_ as a coolant during turning process. By lowering the cutting temperature, friction has been considerably reduced, resulting in an increase in tool life. This improves the surface polish and breakability of chips. They determined that cryogenic machining reduces the cutting force by between 17 and 38%. In addition, they advised replacing traditional coolants with cryogenic CO_2_ while monitoring environmental parameters during cryogenic milling. Furthermore, various researches on tool wear were also undertaken and the effect of coolant utilised was evaluated.

Fang et al. [34] constructed a unique cooling channel to lengthen the tool's life and discovered that increasing coolant flow can enhance machining efficiency and decrease wear resistance. A complete examination of a heat transfer cutting fluid was presented by Gariani et al. [35]. Their unique design was successful in reducing tool flank wear by 46.77 % and minimising fluid consumption by 42 %. Srikant et al. [36] identified nanocoolants as suitable alternatives due to their enhanced thermal conductivity. Due to their heat conductivities, they are beneficial in machining applications. In addition, Srikant et al. [37] noted that 10 % vegetable emulsifier oil can be substituted for conventional machining fluids. Sharma et al. [38] did a review analysis of several lubrication systems, employed in machining operations. Luchesi et al. [39] examined the heat transfer coefficients of cutting fluids through a series of tests. In their study, Ahmed et al. [40] examined the Nusselt number and discovered that in a buoyancy-driven flow, an cavity with square barriers had a quicker rate of heat flow than one with circular barriers. Choi et al. [41] presented a superior heat transfer nanocoolants with enhanced thermal conductivity properties. Chummar and Harish [42,43] analyzed the heat transfer performance of various nanoparticles, such as single-walled carbon nanotubes, aluminium, aluminium oxide, and silver added with water. By modifying the Rayleigh and Reynolds numbers, the research for natural and forced convection heat transfer was carried out. It was revealed that the rate of heat transmission is enhanced by an increase in particle concentration. Murshed et al. [44] discovered that when the temperature increased, so did the thermal conductivity. Moreover, the size, shape, and interfacial layer of the particle all have an impact on thermal conductivity. Moreover, according to Philip et al. [45], the contradictory thermal properties of nanocoolants are mostly due to the complex surface chemistry of nanofluids. Lee et al. [46] investigated the heat transport parameters of a nanofluid containing oxide nanoparticles. Bahmani et al. [47] investigated the heat transfer properties of Al_2_O_3_/H_2_O nanocoolants. Their findings revealed a significant enhancement in both the average Nusselt number and maximum thermal efficiency, with improvements of 32.7 % and 30 %, respectively.

The existing literature review reveals a gap in research concerning the comparison of thermal performance in drilling titanium alloy using ionanofluids containing metallic and carbon nanoparticles. This comparison takes into account factors such as particle volume fraction and inertial force impact on the ionic coolant thermal properties. This gap in knowledge serves as the motivation for the current study. To the best of the author's knowledge, this investigation represents one of the pioneering efforts to explore the turbulent multiphase heat transfer capabilities of ionic liquid coolants enriched with MWCNT nanoparticles in the context of titanium alloy drilling. This could provide insights into how these coolants perform compared to traditional coolants. Using computational fluid dynamics analysis, this study evaluates the heat transfer properties of various ionanofluids utilised as cutting fluids in the drilling process. A computational fluid dynamics-based numerical study is performed in order to comprehend the temperature variation at the contact point of tool and workpiece for various ionanocoolants during a drilling operation. In the simulation setup, the heated workpiece and tool represent the domain, while ionanocoolants are distributed through a nozzle located above the drilling region. Variations in nanoparticle volume fraction and ionanocoolant velocity are used for performing a parametric analysis. The research focuses on analysing flow and heat transfer parameters close to the drilling zone.

## 2 Methodology

### 2.1 Computational domain

**Fig. 1:**
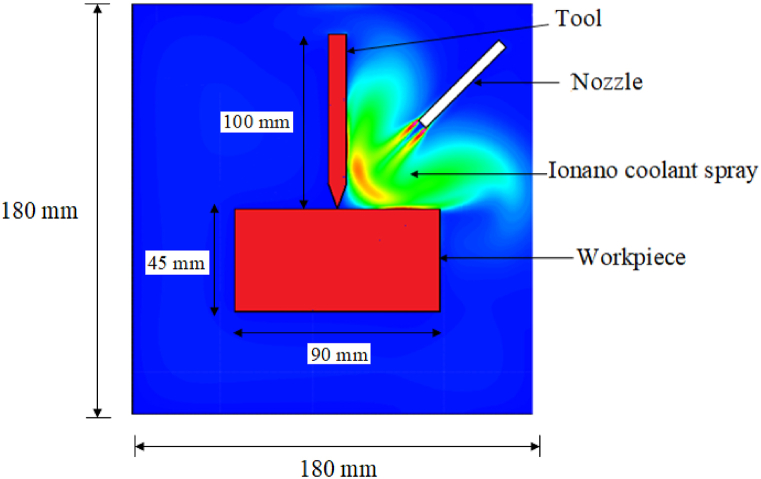
Computational domain of nanoenhanced Ionic liquid spray emerging from the nozzle and directed at the heated drill bit and workpiece.

Fig. 1 depicts the two-dimensional computational domain of the drilling process employing nanoenhanced ionic liquid. The computational domain is 180 × 180 × 180 mm in size, and a nozzle with a diameter of 10 mm and a height of 40 mm is used to spray the drilling zone with ionanocoolants. The drill bit has a diameter of 20 mm and a height of 100 mm, while the width, height and depth of the workpiece are 90 mm, 45 mm and 45 mm, respectively. The workpiece is made of alloy of titanium and the drill bit is considered as Tungsten carbide-cobalt (WC- 10 % Co).

The ionic coolant mixtures is prepared by mixing 10 wt% of [HMIM][BF4] 1-Hexyl-3-methyl-imidazolium tetrafluroburate ionic liquid to mineral oil. Further, nanoparticles of Copper (Cu), Silver (Ag) and MWCNT are dispersed at volumetric percentages forming the various ionanofluid coolants in the study. Particularly, these nanoparticles of copper, silver, and multi-walled carbon nanotubes are considered due to their high thermal conductivity, which improves heat transfer and makes them suitable for a wide range of applications involving heat transfer.

### 2.2 Governing equations and thermophysical properties of nanocoolants

In this investigation, the flow and heat transfer characteristics is assumed as unsteady, three-dimensional turbulent multiphase flows. The realizable k-ε model is one of the most frequently used turbulence models for analysing turbulent flow conditions. The mixture model is used to evaluate the multiphase flow characteristics. There is thermal equilibrium between dispersed Cu/Ag/MWCNT nanoparticles and the base ionic liquid [48,49]. Joule heating and thermal radiations are disregarded. The simulations are performed using Ansys Fluent. The set of governing equations are provided below [22,24]**.**

(1) $$\frac{\partial \left(\rho_{m}\right)}{\partial t}+\nabla \cdot \left(\rho_{m}{\vec{V}}_{m}\right)=0$$

where ρ_m_ and V_m_ represent the mass-averaged values of the variations in density and velocity of the ionanocoolants respectively, and t indicates the dimensional time.

(2) $$\frac{\partial \left(\rho_{m}{\vec{V}}_{m}\right)}{\partial t}+\nabla \cdot \left(\rho_{m}{\vec{V}}_{m}{\vec{V}}_{m}\right)=-\nabla P_{m}+\nabla \cdot \left[\mu_{m}\left(\nabla {\vec{V}}_{m}+\vec{V_{m}^{T}}\right)\right]-\rho_{m}\beta_{m}g\left(T_{h}-T_{c}\right)$$

where P_m_ and μ_m_ indicate the pressure and dynamic viscosity of the ionanocoolant mixture, respectively, and β_m_ and g indicate the coefficient of thermal expansion and acceleration due to gravity.

(3) $$\frac{\partial \left(\rho_{m}C_{pm}T_{m}\right)}{\partial t}+\nabla \cdot \left(\rho_{m}C_{pm}T_{m}\vec{V_{m}}\right)=\nabla \cdot \left[k_{m}\left(\nabla T_{m}\right)\right]$$

where C_pm_, K_m_ and T_m_ indicate the specific heat, thermal conductivity and temperature distribution in the ionanocoolant mixture, respectively.

(4) $$\frac{\partial \left(\varphi_{s}\right)}{\partial t}+\nabla \cdot \left(\varphi_{s}{\vec{V}}_{m}\right)=-\nabla \cdot J_{s}$$

where φ_s_ represents the volume fraction, and J_s_ denotes the mass diffusion flux.

The standard k-ε model is a widely used turbulence model for predicting fluid flow and heat transfer in engineering applications. The model is based on the Reynolds-averaged Navier-Stokes (RANS) equations, which are time-averaged equations of motion for a turbulent flow. The equation of realizable k-ε is provided below:

(5) $$\frac{\partial \left(\rho_{m}k\right)}{\partial t}+\nabla \cdot \left(kV_{m}\right)=\nabla \cdot \left[\left(\mu +\frac{\mu_{t}}{\sigma_{k}}\right)\nabla k\right]+P_{k}+P_{b}-\rho \varepsilon -Y_{m}+S_{k}$$

(6) $$\frac{\partial \varepsilon }{\partial t}+\nabla \cdot \left(\varepsilon V_{m}\right)=\nabla \cdot \left[\left(\mu +\frac{\mu_{t}}{\sigma_{\varepsilon }}\right)\nabla \varepsilon \right]+\rho C_{1}S_{\varepsilon }-\rho C_{2}\frac{\varepsilon^{2}}{k+\sqrt{\upsilon \varepsilon }}+C_{1\varepsilon }\frac{\varepsilon }{k}C_{3\varepsilon }P_{b}+S_{\varepsilon }$$

where k and ε denote the turbulent kinetic energy and rate of energy rate, and P_k_ and P_b_ are the energy generation from shear and the influence of buoyancy force. C_1ε_ and C_2ε_ are empirical constants.

The turbulent viscosity is evaluated as follows:

(7) $$\mu_{t}=\rho C_{\mu }\frac{k^{2}}{\varepsilon }$$

The production of turbulent kinetic energy term is evaluated as follows:

(8) $$P_{k}=-\rho u_{i}^{\prime }u_{j}^{\prime }\frac{\partial u_{j}}{\partial x_{i}}$$

The thermal buoyancy effects are evaluated as follows:

(9) $$P_{b}=\beta g_{i}\frac{\mu_{t}}{{\mathrm{Pr}}_{t}}\frac{\partial T}{\partial x_{i}}$$

The empirical constants in the dissipation equation are considered as

(10) $$C_{1\varepsilon }=1.44\text{,}C_{2\varepsilon }=1.92\text{,}C_{\mu }=0.09\text{,}\sigma_{k}=1\text{,}\sigma_{\varepsilon }=1.3$$

The following empirical models are used to calculate the thermophysical properties of the various ionanofluid coolants that are chosen for this study. In this study, the ionic liquid is used as the base fluid in which the nanoparticles are dispersed. To comprehensively model the behavior of this nano-enhanced ionic liquid, the properties of both the ionic fluid and the nanoparticles are considered. Correlations given by Eqs. (11), (12), (13), (14) account for the combined effect of the nanoparticles and ionic fluid on the thermal conductivity, density, and specific heat capacity of the mixture. The empirical models used to evaluate the thermophysical properties of an ionanocoolant mixture are valid up to a nanoparticle volumetric concentration of 8 %. In this research, the nanoparticle volumetric concentration is therefore varied between 2 % and 8 %. Table 1 shows the thermophysical properties of the base ionic liquid and nanoparticles [50,51]. The thermophysical properties of the base ionic liquid with Cu, Ag, and MWCNT nanoparticles added at various nanoparticle volume fractions are shown in Table 2, Table 3, Table 4.

**Table 1:** Ionic liquid and nanoparticles physical properties [50,51].

Name	Specific heat (J/kg-K)	Density (kg/m^3^)	Thermal conductivity (W/m-K)	Dynamic viscosity, (*N*-s/m^2^)
[HMIM][BF_4_]	2266	1145.4	0.166	0.25
Cu	385	8933	401	–
Ag	235	10,500	429	–
MWCNT	711	2100	3000	–

**Table 2:** Thermophysical properties of the base ionic liquid added with Cu nanoparticles.

Name	Specific heat (J/kg-K)	Density (kg/m^3^)	Thermal conductivity (W/m-K)	Dynamic viscosity, (*N*-s/m^2^)
[HMIM][BF_4_] + 2 % Cu	2228.38	1301.152	0.172767	0.262951
[HMIM][BF_4_] + 4 % Cu	2190.76	1456.904	0.179815	0.276861
[HMIM][BF_4_] + 6 % Cu	2153.14	1612.656	0.187164	0.291824

**Table 3:** Thermophysical properties of the base ionic liquid added with Ag nanoparticles.

Name	Specific heat (J/kg-K)	Density (kg/m^3^)	Thermal conductivity (W/m-K)	Dynamic viscosity, (*N*-s/m^2^)
[HMIM][BF_4_] + 2 % Ag	2225.38	1332.492	0.172767	0.262951
[HMIM][BF_4_] + 4 % Ag	2184.76	1519.584	0.179817	0.276861
[HMIM][BF_4_] + 6 % Ag	2144.14	1706.676	0.187165	0.291824

**Table 4:** Thermophysical properties of the base ionic liquid added with MWCNT nanoparticles.

Name	Specific heat (J/kg-K)	Density (kg/m^3^)	Thermal conductivity (W/m-K)	Dynamic viscosity, (*N*-s/m^2^)
[HMIM][BF_4_] + 2 % MWCNT	2234.9	1164.492	0.172774	0.262951
[HMIM][BF_4_] + 4 % MWCNT	2203.8	1183.584	0.179831	0.276861
[HMIM][BF_4_] + 6 % MWCNT	2172.7	1202.676	0.187188	0.291824

Thermal conductivity of ionanocoolants with dispersed metallic spherical nanoparticles is calculated using the following correlation [20,21,52,53], as:

(11) $$\frac{k_{m}-k_{bf}}{k_{bf}}=\frac{k_{np}}{k_{bf}}\left(1+c\frac{u_{np}d_{np}}{\alpha_{bf}}\right)\frac{d_{bf}}{d_{np}}\frac{\varphi }{1-\varphi }$$

where k_m_, k_f_ and k_np_ denotes the thermal conductivity of the nanocoolant mixture, base fluid and nanoparticle respectively.

Similarly, the effective thermal conductivity of MWCNT ionanocoolants dispersed with cylindrical nanoparticles are evaluated using the following relations:

(12) $$\frac{k_{m}}{k_{bf}}=\frac{k_{np}+\left(n-1\right)k_{bf}+\left(n-1\right)\varphi \left(k_{np}-k_{bf}\right)}{k_{np}+\left(n-1\right)k_{bf}-\varphi \left(k_{np}-k_{bf}\right)}$$

where emprical shape factor is evaluated as $n=\frac{3}{\psi },\psi =\frac{\pi^{1/3}{\left(6v_{p}\right)}^{2/3}}{A_{p}}$.

The viscosity of the ionanofluids is calculated as [22,24].

$$\mu_{eff}=\frac{\mu_{bf}}{{\left(1-\varphi \right)}^{2.5}}$$

The model to calculate ionanofluid density is provided below.

(13) $$\rho_{m}=\varphi \rho_{np}+\left(1-\varphi \right)\rho_{bf}$$

where ρ_m_, ρ_f_ and ρ_np_ denotes the density of the nanocoolant mixture, base fluid and nanoparticle respectively.

The model to calculate specific heat of ionanofluids is provided below.

(14) (ρc)pm=(1−φ)(ρcp)bf+φ(ρcp)np

The following boundary conditions are used:

(15) $$\text{Nozzle}\;\text{Inlet}:v=v_{\infty },T=T_{\infty }$$

Workpiece tool interface:

(16) $$T=1100K,u=0\text{,}v=0\text{,}k=0\text{,}\frac{\partial \varepsilon }{\partial n}=0$$

Walls of computational domain:

(17) $$u=0\text{,}v=0\text{,}k=0\text{,}\frac{\partial \varepsilon }{\partial n}=0\text{,}\frac{\partial T}{\partial n}=0$$

The nozzle is specified with a velocity inlet boundary condition and the workpiece and tool interface is specified with a temperature of 1100 K. The governing equations are discretized using finite volume method. A pressure-velocity scheme of SIMPLE is adopted and the diffusion term in the momentum, energy equation, turbulent kinetic energy and dissipation are discretized using second order discretization technique and the residuals are set to a value of 10^−6^.

### 2.3 Grid independence study

**Fig. 2:**
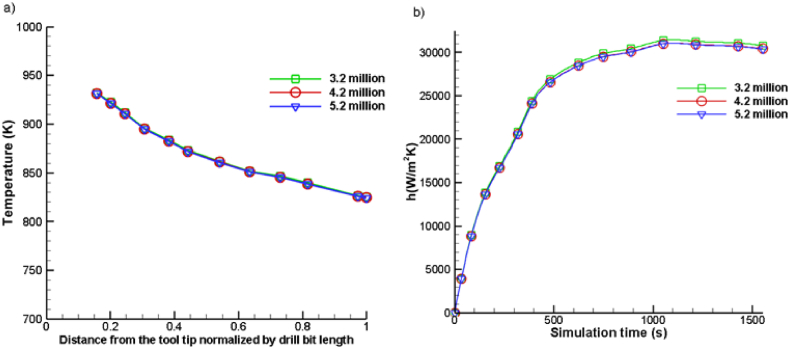
Grid Independence Test performed using mesh of three different sizes and the results are plotted for: a) Temperature Profile and b) Heat Transfer Coefficient.

Fig. 2 illustrates the investigation of grid independence, which was carried out using three distinct mesh sizes: 5.2 million, 4.2 million, and 3.2 million cells. This investigation focused on the temperature profile at the tool tip, specifically for an ionic liquid mixed with MWCNT nanoparticles at a volume proportion of 8 %. In Fig. 2(a), it can be observed that the temperature of the tool tip decreases as the distance from the tool tip increases. By zooming in, the temperature distribution differences between the mesh sizes become evident. The temporal variation of the heat transfer coefficient for the three mesh sizes is presented in Fig. 2(b), and it is observed that as the mesh size increases from 4.2 million to 5.2 million, the deviations observed become significantly smaller. Consequently, a mesh size of 4.2 million cells is employed for this computational investigation.

## 3 Results and discussion

### 3.1 Validation

**Fig. 3:**
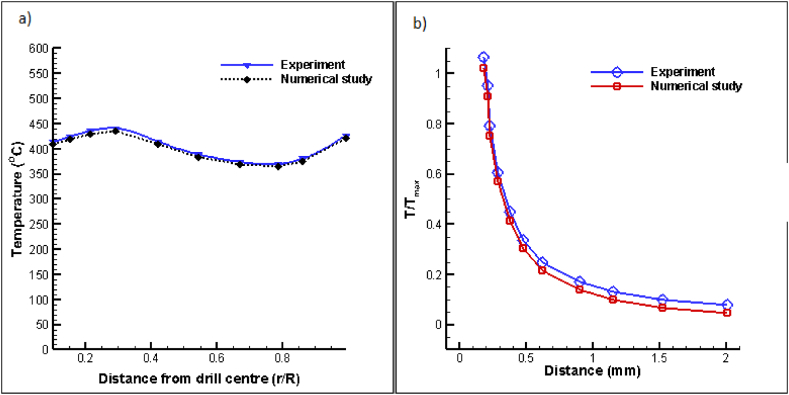
Comparative validation graphs between the current study with a) Temperature variation from drill bit center compared with Zhu et al. [54] and b) Temperature fluctuations from the center of the drill bit compared with Khan et al. [55].

For the validation of the computational studies conducted in this research project, Fig. 3 displays the results in comparison with previously published literature. In Fig. 3(a), a comparison is made between the numerical results presented here and the benchmark results of Zhu et al. [54]. In the experiments carried out to establish the temperature profile of the drill bit while drilling aluminium at a flow rate of 60 mL/h with minimal quality lubrication (MQL), it was observed that the temperature at the center of the drill is higher than that towards the outer corner. The heat generated at the center of the drill during the drilling operation accumulates since it cannot dissipate immediately, leading to an elevated temperature close to the drill's center. Khan et al. [55] examined thermal performance in drilling operations using nanoparticles made of clay, and the experimental validation plots are shown in Fig. 3(b). Their findings suggested that incorporating clay nanoparticles into coolants increases the thermal conductivity of the resulting composition, rendering the components more tolerant of high temperatures. Consequently, they analyzed the temperature change relative to the distance from the drill bit's point. The observations indicated that the temperature of the drill tip decreases as the distance increases. Similar trends can be observed in the present research. The figures depict the temperature variation predicted between the present numerical model and experimental results are in accordance with the benchmark findings reported in literature.

### 3.2 Transient flow and heat transfer characteristics

By studying the velocity, temperature, pressure, and kinetic energy contours, the flow and.

**Fig. 4:**
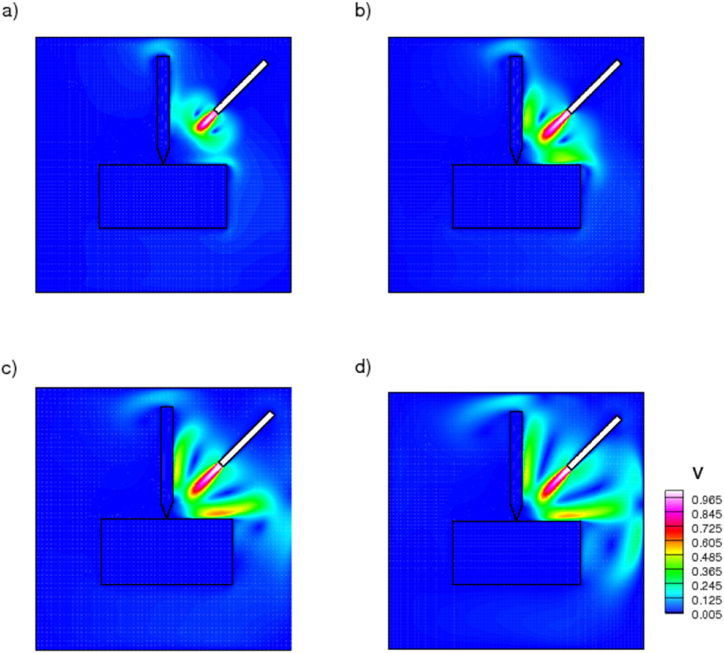
Velocity contours of nanoenhanced [HMIM][BF_4_] coolant sprayed from nozzle at various time, a) 100, b)400, c) 600, d)1000 s.

Thermal performance of a nanoparticle-dispersed ionic liquid are explored. Fig. 4 depicts the velocity contours of [HMIM][BF4] nanoenhanced ionic liquid containing copper nanoparticles. The contour depicts the transient variation of velocity distribution at time steps 100, 400, 600, and 1000 s. When nanoparticles are dispersed in ionic liquids at various volumetric fractions, the thermal conductivity of the resulting ionanocoolants increases. The ionic nanocoolant spray is observed to originate from the nozzle and propagate towards the drill point-contact zone. Initially, the velocity of the coolant is greatest near the orifice, and it gradually approaches the drilling zone as time passes. As a result of the enhanced thermal conductivity, coolant fluid sprayed at the drilling zone can absorb more heat due to forced convection. It is evident that the spraying velocity of the coolant increases with time and that it diffuses heat at the interface between the tool and the chip. This contour enables us to understand the flow and interaction between ionanocoolant and the drilling zone. This research broadens the scope of conventional coolant fluids currently utilised in industry.

**Fig. 5:**
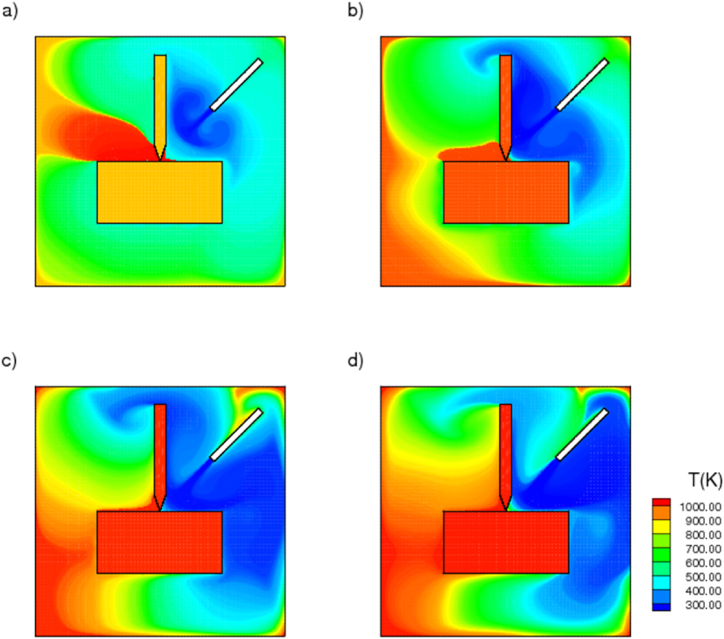
Temperature contours of nanoenhanced [HMIM][BF_4_] coolant sprayed from nozzle at various time, a) 100, b)400, c) 600, d)1000 s.

Fig. 5 illustrates the transient temperature variation when ionanofluid is sprayed over the interface between the tool and the workpiece at 100, 400, 600, and 1000 s intervals. Noticeably, a stream of cold fluid is discharged from the nozzle, covering the cutting area and dissipating the heat generated by drilling. Indicated as well is the change in coolant temperature as it travels from the nozzle region to the drilling zone. It has been observed that the temperature is initially relatively low near the nozzle and that the cold stream progressively reduces the temperature in the regions surrounding the workpiece and the drill bit. The temperature distribution within the computational domain decreases considerably over time, and the ionic liquid distributed with copper nanoparticles at a volume fraction of 2 % is notably effective at removing the accumulated heat. This reduces the frictional heat generated between the tool and the workpiece, thereby extending the life of the drilling tool. Since the computational domain is closed and has walls at its extremities, the heat carried away by the ionanocoolant is circulated within the cavity as a result of forced convection. Moreover, the walls are treated as adiabatic boundary conditions, and high temperatures are observed in the upper right corners of the computational domain.

**Fig. 6:**
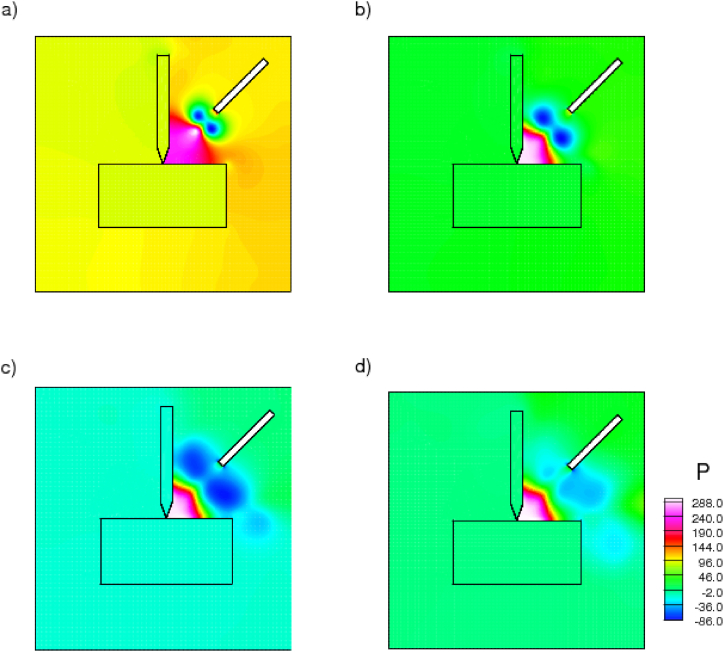
Pressure contours of nanoenhanced [HMIM][BF_4_] coolant sprayed from nozzle at various time, a) 100, b)400, c) 600, d)1000 s.

Fig. 6 depicts the contours of pressure variation for the ionanocoolant fluid at time intervals of 100, 400, 600, and 1000 s. It is evident from the contours that the initial pressure in the drilling zone is greater because of the extremely high temperature. With time, the cold fluid stream originating from the nozzle diffuses the heat and reduces the pressure distribution at the drill point-contact zone. Positive values of initial high pressure distribution near the interface of the tool and workpiece are the result of heat generation, whereas negative values represent the pressure of the coolant discharge. Clearly, the pressure of the coolant reduces the pressure resulting from high temperature and eliminates heat in the drilling zone.

**Fig. 7:**
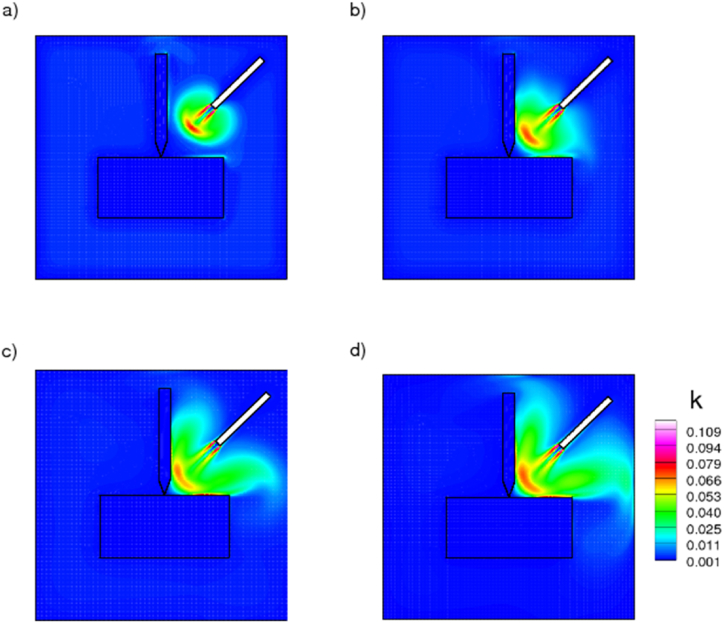
Kinetic energy contours of nanoenhanced [HMIM][BF_4_] coolant sprayed from nozzle at various time, a) 100, b)400, c) 600, d)1000 s.

**Fig. 8:**
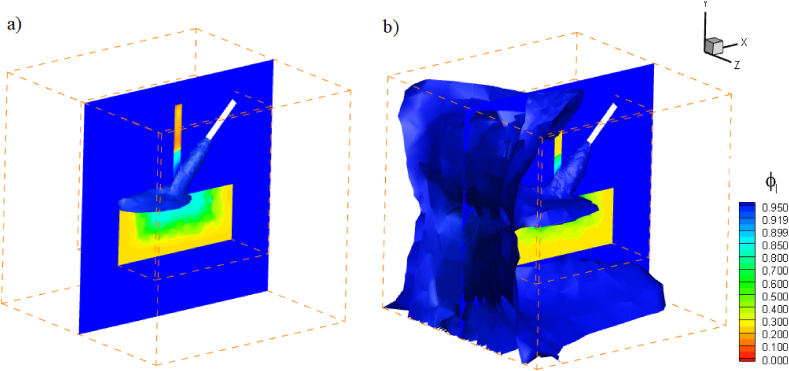
Liquid fraction isosurfaces of nanocoolant jet sprayed from nozzle at various time, a) 400, b) 1000 s.

Fig. 7 depicts the kinetic energy contours of the coolant spray. Initial kinetic energy values are high in regions where coolants are sprayed near to the nozzle, and the inertial force of the coolant sprayed at a high velocity disperses heat in the drilling zone. Coolant sprayed at high velocity increases the inertial force and reduces the rate of convective heat transfer in the regions surrounding the drill bit and workpiece. Including nanoparticles with ionic liquid enhances the Brownian motion and thermophoresis properties of the ionic mixture, hence controlling the heat generated during drilling. In Fig. 8, the progression of the liquid fraction of the ionanocoolant spray is illustrated as it emanates from the nozzle. The coolant effectively absorbs the heat produced by the drill bit and workpiece, thereby ensuring the maintenance of an optimal drilling temperature.

**Fig. 9:**
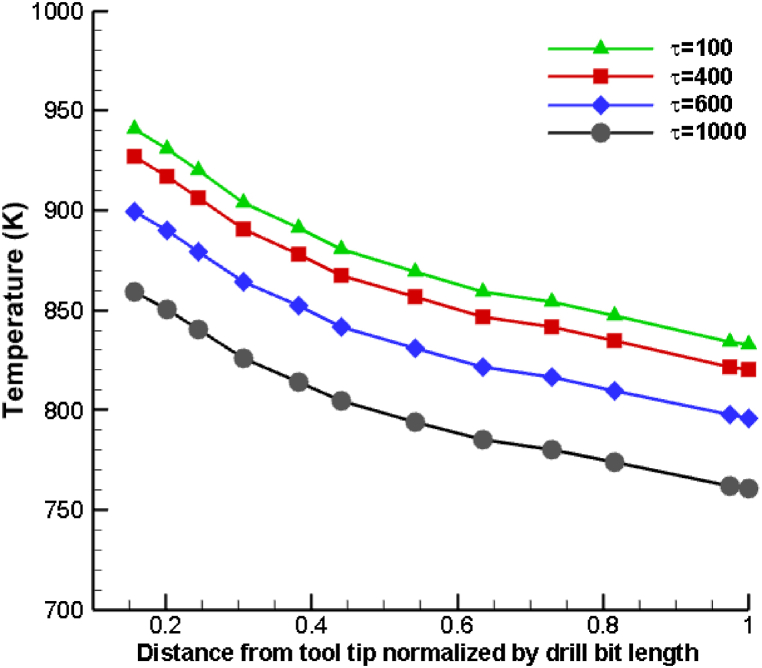
Comparison of temperature variation of [HMIM][BF_4_] + 2 % Cu nanoparticles being used as coolant fluids at different time.

Fig. 9 depicts the dynamic fluctuation of temperature variation of the base ionic liquid distributed with 2 % volume fraction of copper nanoparticles in the normal direction of the tool in the drilling zone at 100, 400, 600, and 1000s intervals. Apparently, the evolution of time led to a decline in temperature levels. Due to the nanoparticle suspension, the thermal conductivity of the ionic fluid has increased, resulting in a decrease in the temperature distribution. The primary reason for this is that nanoparticles exhibit a substantially greater thermal conductivity compared to the ionic fluid. Increased thermal conductivity of an ionic fluid enhances heat transport and temperature diffusion properties. Similarly, as time progresses, the fluid mixture's specific heat rises and more heat is lost. In addition, as time progresses, a greater quantity of ionic coolant mixture reaches the drilling zone, and the inertial forces of the spray enhance the forced convection-induced heat transfer in the drilling zone. During the drilling operation, the suitable temperature is maintained by the ionic liquid combination as the drilling temperature decreases linearly with time.

### 3.3 Effect of nanoparticle types and volume fraction

**Fig. 10:**
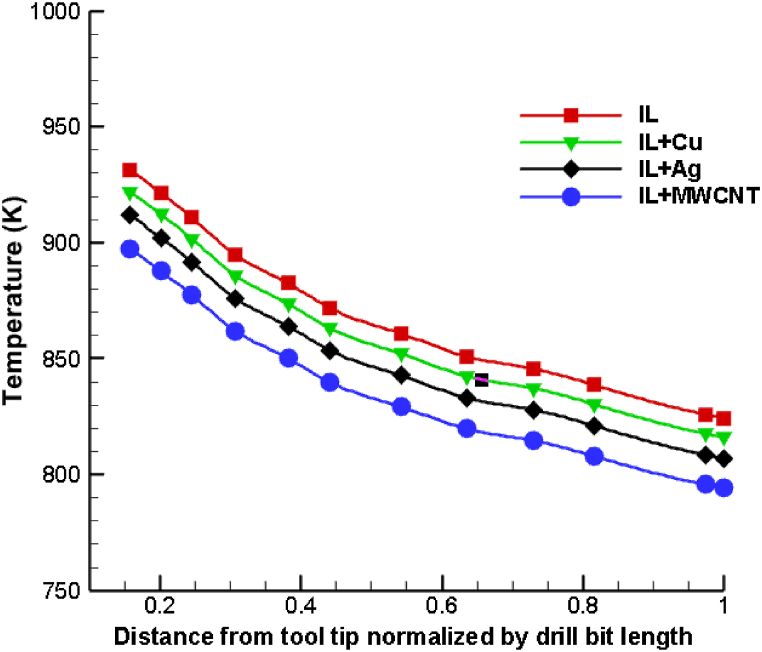
Comparison of temperature distribution of pure ionic liquid with various nanoenhanced ionic liquid.

Fig. 10 illustrates the temperature variations in the drilling zone relative to the normal distance from the drill center for pure ionic liquid and nanoenhanced ionic liquid mixed with copper, silver, and MWCNT nanoparticles at a volume fraction of 4 %. The graph demonstrates that when nanoparticles are added to the base ionic liquid, the maximal temperature decreases significantly compared to the base ionic liquid. In addition, the thermal performance of the MWCNT-dispersed drilling fluid is superior to that of copper and silver. This is a result of the enhanced thermophysical properties of the coolants used in ionano drilling, such as increased thermal conductivity and specific heat capacity. Copper nanoparticles added to ionic liquid reduce the temperature of drilling by 10.66 %, silver nanoparticles by 12.36 %, and MWCNT nanoparticles by 15.13 %. Thus, compared to copper and silver nanoparticles, the combination of ionic liquid dispersed with MWCNT results in superior performance and is a superior coolant. This research could be broadened by investigating hybrid ionanofluid coolant pairs as well as varying the shape and size of nanoparticles and analysing their thermal performance when used as a cutting fluid in a variety of drilling processes.

**Fig. 11:**
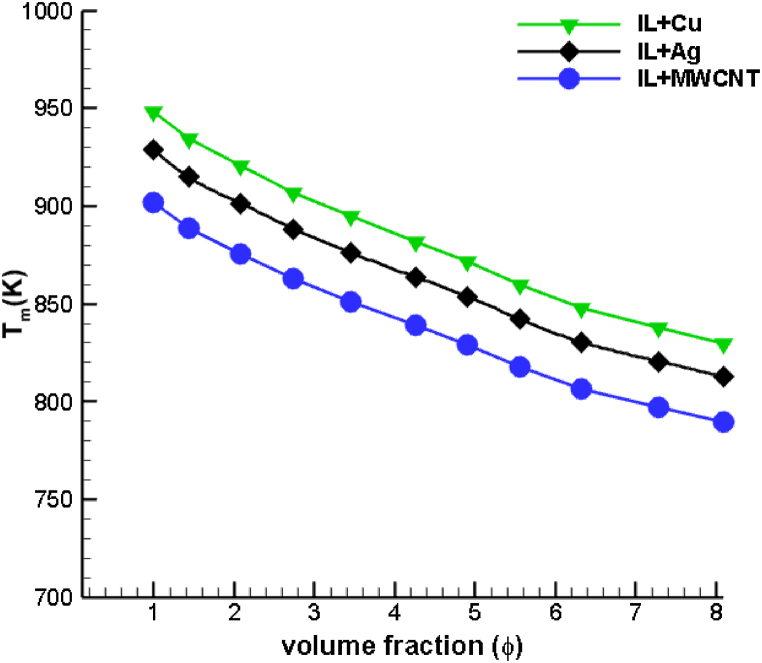
Comparison of maximum temperature variation of [HMIM][BF_4_] dispersed with Cu, Ag and MWCNT nanoparticles for various volume fractions.

In Fig. 11, a comparison is presented among different ionanofluid cooling agents, showcasing the variation in peak temperature attained at the tool tip with increasing concentrations of dispersed copper, silver, and MWCNT nanoparticles. The concentration levels ranged from 1 % to 8 %, and it was observed that the maximum temperature in the drilling zone exhibited a consistent linear decrease as the concentration of nanoparticles increased, regardless of the nanoparticle combinations. In contrast to other nanoparticle combinations, MWCNT ionic coolants exhibited a more uniform temperature distribution. The decrease in temperature is consistent with the hypothesis that nanoparticles enhance cooling by accelerating the rate of heat transfer. Increasing the volumetric concentration of copper nanoparticles results in an 11.68 % reduction in the extent of the maximum temperature distribution, whereas silver and MWCNT nanoparticles reduce the maximum temperature distribution by 13.98 % and 16.28 %, respectively. When increasing volume fractions of MWCNT nanoparticles are dispersed in an ionic liquid, the drilling temperature is drastically reduced. Coolants dispersed with carbon nanotubes are therefore more efficient heat transmission fluids than those dispersed with nanoparticles composed of metals and metal oxides.

**Fig. 12:**
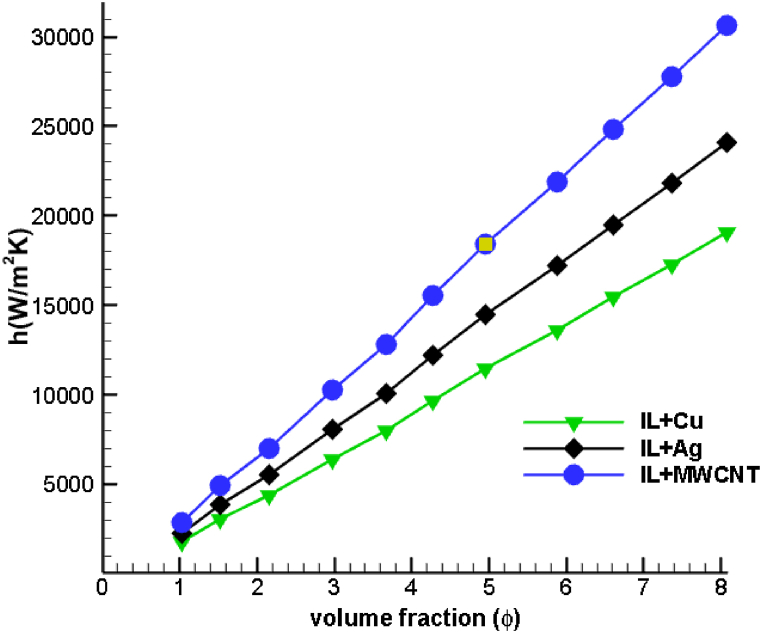
Comparison of heat transfer coefficient of [HMIM][BF_4_] dispersed with Cu, Ag and MWCNT nanoparticles for various volume fractions.

Fig. 12 depicts the evolution of the heat transfer coefficient with the dispersion of various nanoparticles at varying volume fractions in ionic liquid. It is interesting to note that the variation of heat transfer coefficient between the three nanoparticles is negligible at 1 % volume fraction, indicating a slower rate of Brownian motion and thermophoresis. However, at higher volume fractions, a significant difference is observed in the values of heat transfer coefficient between the three ionanofluid mixtures. In general, the heat transfer coefficient rises as particle volume fraction increases. It has been concluded that the heat transfer coefficient of MWCNT ionanofluid coolants is greater than that of copper and silver ionic liquid combinations. This is supported by the fact that the thermal conductivity and specific heat value of MWCNT are greater than those of copper and silver. Therefore, the augmentation in volumetric concentration increases the heat exchange between the drilling zone and the nanofluid. Compared to ionanofluid dispersed with copper, silver ionanofluid coolant enhances the heat transfer coefficient by 23.15 % for a higher particle volume fraction of 8 %, whereas MWCNT dispersed ionic coolant boosts it by 66.5 %. This verifies the hypothesis that ionic liquids dispersed with MWCNT nanoparticles enhance heat transfer.

**Fig. 13:**
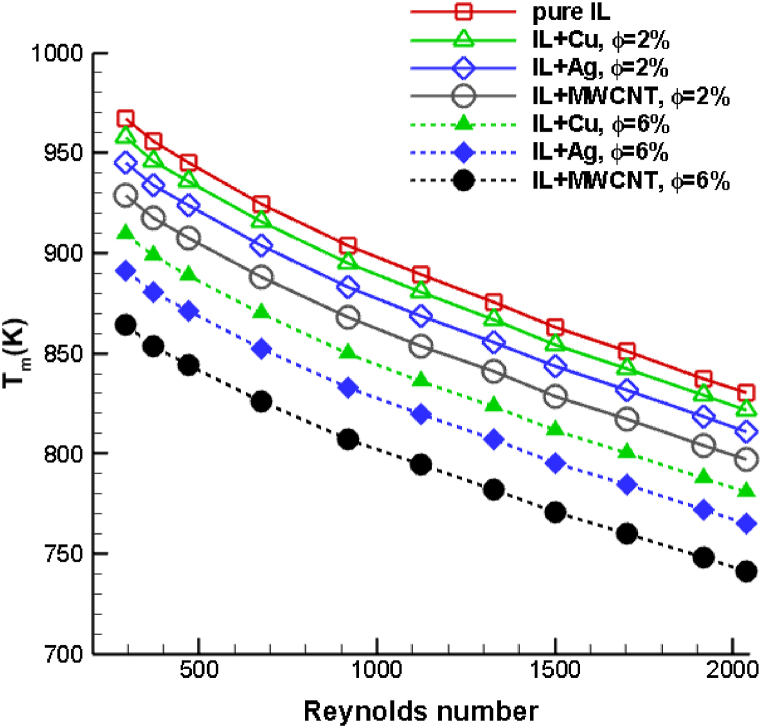
Comparison of maximum temperature of [HMIM][BF_4_] dispersed with Cu, Ag and MWCNT nanoparticles for various Reynolds number and volume fractions.

Fig. 13 illustrates the maximum temperature in the drilling zone for three distinct ionanofluids utilizing an identical 8 % volumetric concentration of nanoparticles. The range of Reynolds numbers employed varies from 250 to 2000. It is noticeable that when the Reynolds number increases, the temperature curves for all ionanofluid combinations decreases linearly. This is due to the fact that an increase in Reynolds number enhances the spraying velocity of the coolant, which in turn boosts the inertial force of the coolant, enabling a significant quantity of heat to be diffused from the drilling zone. The ionanocoolant mixed with MWCNT nanoparticles demonstrated a greater reduction in cutting temperature than the mixture of copper and silver ionic liquids, and the drilling temperature drops as the particle volume fraction increases. The MWCNT ionic coolant mixture with an 8 % particle volume fraction reduces the drilling temperature of pure ionic liquid by 25.64 %

Within Table 5, Table 6, the heat transfer coefficient of diverse ionanofluids is presented, exhibiting varying volumetric proportions of nanoparticles and Reynolds numbers. The heat transfer coefficient is observed to increase as a function of both volume fraction and Reynolds number. The augmentation of volumetric ratio leads to an elevation in the rate of heat transfer as a consequence of the incorporation of additional nanoparticles, thereby enhancing the thermal performance of the ionanofluid. Similarly, the increase in Reynolds number increases the inertial forces and dissipates more heat from the drilling zone.

**Table 5:** Heat transfer coefficient of various ionanofluids by varying volumetric fraction of nanoparticles.

φ (%)	IL + Cu h (W/m^2^K)	IL + Ag h (W/m^2^K)	IL + MWCNT h (W/m^2^K)
1	1850	2000	2300
2	4300	5700	7800
3	6550	8000	10,200
4	9350	11,750	15,820
5	11,500	14,000	18,000
6	13,350	15,800	22,150

**Table 6:** Heat transfer coefficient of various ionanofluids by varying Reynolds Number.

*Re*	IL h (W/m^2^K)	IL+2 % Cu h (W/m^2^K)	IL+ 2 % Ag h (W/m^2^K)	IL+2%MWCNT h (W/m^2^K)	IL+6%Cu h (W/m^2^K)	IL+6%Ag h (W/m^2^K)	IL+6%MWCNT h (W/m^2^K)
140	5850	6500	7100	7500	7850	8360	8500
260	9500	10,250	11,450	11,500	11,550	11,940	13,100
465	13,580	14,830	15,500	16,000	16,540	17,000	14,870
670	18,200	19,800	20,500	21,675	22,236	23,410	25,000
950	22,500	24,000	25,430	26,750	28,970	29,860	32,485
1200	28,000	30,850	31,860	37,860	33,487	36,700	39,540
1500	33,480	34,500	36,790	38,220	37,430	41,510	42,800
1700	35,450	37,850	42,800	43,400	44,320	47,820	51,500
1900	37,800	41,800	44,970	47,135	48,120	51,150	54,910
2000	40,100	43,100	46,780	49,745	51,530	52,400	57,630

Within Table 5, Table 6, the heat transfer coefficient of diverse ionanofluids is presented, exhibiting varying volumetric proportions of nanoparticles and Reynolds numbers. The heat transfer coefficient is observed to increase as a function of both volume fraction and Reynolds number. The augmentation of volumetric ratio leads to an elevation in the rate of heat transfer as a consequence of the incorporation of additional nanoparticles, thereby enhancing the thermal performance of the ionanofluid. Similarly, the increase in Reynolds number increases the inertial forces and dissipates more heat from the drilling zone.

**Fig. 14:**
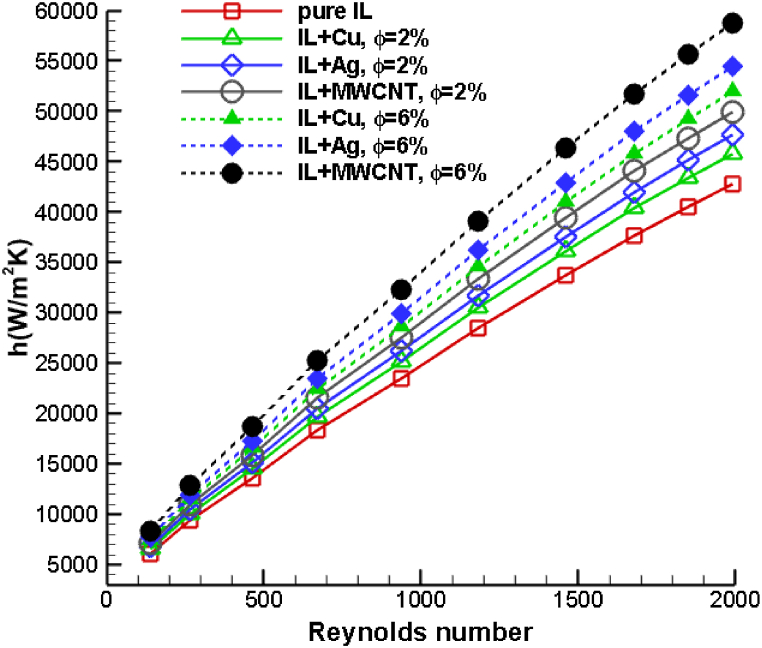
Comparison of heat transfer coefficient of [HMIM][BF_4_] dispersed with Cu, Ag and MWCNT nanoparticles for various Reynolds number and volume fractions.

In Fig. 14, a comparative analysis is presented, showcasing the average heat transfer coefficient across different Reynolds numbers and volume concentrations for all the ionanofluid combinations considered in the study. It is observed that the convective heat transfer coefficient has enhanced as the Reynolds number has increased. In addition, as the volumetric concentration of nanoparticles increases, the convective heat transfer coefficient also increases. At lower Reynolds numbers, however, the effect of nanoparticle introduction on the heat transfer coefficient is insignificant. The rise in Reynolds number value boosts the inertial force of the ionanocoolant spray, and the ionic liquid mixed with MWCNT nanoparticles exhibits the maximum heat transfer coefficient. For a higher volume fraction of 8 %, the copper, silver, and MWCNT ionanofluid mixture increases the average heat transfer coefficient of pure ionic coolant by 35.14 %, 47.42 %, and 62.75 %, respectively. Hence, it can be inferred that MWCNT-dispersed ionic coolants exhibit superior heat transfer characteristics and are recommended as suitable drilling coolants. In this investigation, it was established that coolants dispersed with MWCNT nanoparticles are more effective heat transfer fluids than those dispersed with metal and metal–oxide nanoparticles. These results are consistent with prior research in the field and support our research objectives to evaluate the heat transfer properties of various ionanofluids used as drilling cutting fluids.

## 4 Conclusions

In this study, a numerical investigation is conducted to compare the temperature variation on the tool-to-workpiece contact during drilling with various ionanocoolants. A computational domain of dimension 180 × 180 × 220 mm is considered, along with a drill bit of diameter 10 mm. Using a nozzle with a 10 mm diameter, ionanocoolants are sprayed into the drilling zone. The drill bit is composed of tungsten carbide-cobalt (WC- 10 % Co), while the material of the workpiece is titanium alloy Ti–6Al–4V. Mixing 10 wt% of 1-Hexyl-3-methyl-imidazolium tetrafluroburate ionic liquid with mineral oil produces ionic coolant mixtures. In addition, nanoparticles of Cu, Ag, and MWCNT are dispersed at various volumetric percentages to form various ionanofluid coolants. By varying the nanoparticle concentration percentage and ionanocoolant velocity, the heat transfer performance of a variety of ionanocoolants is compared. For various volume fractions and Reynolds numbers, the parameters including tool temperature and coefficient of heat transfer are examined. According to the results, ionanofluids with greater thermal conductivity will demonstrate superior thermal behavior. The following is a summary of this investigation's findings.

• It is observed that the ionic nanocoolant mist is released from the nozzle and directed towards the drill bit-workpiece interface. Initially, the coolant's velocity is greatest close to the orifice, and as time passes, it approaches the drilling region. It is apparent that the spraying velocity of the coolant increases over time and that it disperses heat at the tool-chip interface. The results help us to identify the flow and interaction of ionanocoolant with the drilling zone. The temperature of the drilling tool and the workpiece drops linearly as the volume fraction of added nanoparticles increases. Coolant sprayed at high velocity enhances inertial force and decreases convective heat transfer in regions surrounding the drill bit and workpiece. The addition of nanoparticles to ionic liquid improves the Brownian motion and thermophoresis properties of the ionic mixture, hence regulating the heat generated during drilling.
• Copper nanoparticles added to ionic liquid decrease drilling temperature by 10.66 %, silver nanoparticles by 12.36 %, and MWCNT nanoparticles by 15.13 %.
• The increase in volume fraction consequently enhances the heat exchange between the drilling zone and the nanofluid. Compared to ionanofluid dispersed with copper, silver ionanofluid coolant increases the heat transfer coefficient by 23.15 % for a higher particle volume fraction of 8 %, whereas MWCNT dispersed ionic coolant increases it by 66.55 %. This study shows that ionic liquids dispersed with MWCNT nanoparticles improve heat transfer.
• The ionanocoolant combined with MWCNT nanoparticles reduced the cutting temperature more than the mixture of copper and silver ionic liquids, and the drilling temperature decreases as the particle volume fraction increases. The higher particle volume fraction of the MWCNT ionic coolant combination reduces the drilling temperature of pure ionic liquid by 25.64 %
• .For a higher volume fraction of 8 %, the copper, silver, and MWCNT ionanofluid mixture increases the average heat transfer coefficient of pure ionic coolant by 35.14 %, 47.42 %, and 62.75 %, respectively. Hence, it can be inferred that MWCNT-dispersed ionic coolants exhibit superior heat transfer characteristics and are recommended as suitable drilling coolants.

In conclusion, nanoparticle-enhanced ionic liquid drilling coolants demonstrate immense potential in enhancing heat transfer during drilling processes. The incorporation of nanoparticles in ionic liquid-based coolant systems has shown promising results, leading to improved thermal performance. These advanced cooling solutions offer benefits such as enhanced heat dissipation, reduced tool wear, and improved drilling efficiency. With their ability to effectively manage heat transfer, nanoparticle-enhanced ionic liquid coolants hold great promise in various industrial applications, including metalworking, drilling, and precision engineering. Further research and development in this area can pave the way for the widespread adoption of these innovative cooling solutions, ultimately contributing to more efficient and sustainable manufacturing processes.

## Data availability

Data will be made available on request.

## Funding details

There are no funding sources associated with this work.

## Disclosure of conflict of interest

The authors declare that they have no conflict of interest.

## Ethical approval

Not required.

## Additional information

No additional information is available for this paper.

## CRediT authorship contribution statement

**B. Srivathsan:** Conceptualization, Data curation, Formal analysis, Methodology, Validation, Writing – original draft, Visualization. **Thaniarasu G:** Conceptualization, Data curation, Formal analysis, Methodology, Validation, Writing – original draft, Visualization. **K. Vishnu Ram:** Data curation, Formal analysis, Methodology, Writing – original draft, Visualization. **Harish R:** Conceptualization, Methodology, Project administration, Resources, Software, Supervision, Writing – original draft, Writing – review & editing.

## Declaration of competing interest

The authors declare that they have no known competing financial interests or personal relationships that could have appeared to influence the work reported in this paper.

## References

[1] Sultana M.N., Dhar N.R. (2023). Comparative evaluation and sensitivity analysis of multi-modelling and optimization of milling Ti–6Al–4V alloy with high-pressure coolant jets. Heliyon.

[2] Girinon M., Karaouni H., Masciantonio U., Lefebvre F., Jourden E., Valiorgue F., Rech J., Feulvarch E. (2019). Risks related to the lack of lubrication on surface integrity in drilling. Heliyon.

[3] Di C., Dinghua Z., Baohai W., Ming L. (2017). An investigation of temperature and heat partition on tool-chip interface in milling of difficult-to-machine materials. Procedia CIRP.

[4] Baohai W., Di C., Xiaodong H., Dinghua Z., Kai T. (2016). Cutting tool temperature prediction method using analytical model for end milling. Chin. J. Aeronaut..

[5] Aparicio S., Atilhan M., Karadas F. (2010). Thermophysical properties of pure ionic liquids: review of present situation. Ind. Eng. Chem. Res..

[6] Qu J., Truhan J.J., Dai S., Luo H., Blau P.J. (2006). Ionic liquids with ammonium cations as lubricants or additives. Tribol. Lett..

[7] Gupta M., Singh V., Kumar R., Said Z., Z (2017). A review on thermophysical properties of nanofluids and heat transfer applications. Renew. Sustain. Energy Rev..

[8] Ouabouch O., Kriraa M., Lamsaadi M. (2021). Stability, thermophsical properties of nanofluids, and applications in solar collectors: a review. AIMS Materials Science.

[9] Yu W., Choi S.U.S. (2003). The role of interfacial layers in the enhanced thermal conductivity of nanofluids: a renovated maxwell model. J. Nanoparticle Res..

[10] Visconti P., Primiceri P., Costantini P., Colangelo G., Cavalera G.G. (2016). Measurement and control system for thermosolar plant and performance comparison between traditional and nanofluid solar thermal collectors. Int. J. Smart Sens. Intell. Syst..

[11] Chakraborty S., Panigrahi P.K. (2020). Stability of nanofluid: a review. Appl. Therm. Eng..

[12] Ghadimi A., Saidur R., Metselaar H.S.C. (2011). A review of nanofluid stability properties and characterization in stationary conditions. Int. J. Heat Mass Tran..

[13] Abdulhameed A., Halim M.M., Halin I.A. (2023). Simulation and experimental validation of the interplay between dielectrophoretic and electroosmotic behavior of conductive and insulator particles for nanofabrication and lab-on-chip applications. Colloids Surf., A: Physicochem. Eng..

[14] Abdulhameed A., Mohtar M.N., Hamidon M.N., Halin I.A. (2022). Assembly of long carbon nanotube bridges across transparent electrodes using novel thickness-controlled dielectrophoresis. Electrophoresis.

[15] Abdulhameed A., Halim M.M. (2023). Electrical and thermal conductivity enrichment by carbon nanotubes: a mini-review. Emergent mater.

[16] Abdulhameed A., Mahnashi Y. (2023). Fabrication of carbon nanotube/titanium dioxide nanomaterials-based hydrogen sensor using novel two-stage dielectrophoresis process. Mater. Sci. Semicond. Process..

[17] Hossain R., Azad A.K., Hasan M.J., Rahman M.M. (2022). Radiation effect on unsteady MHD mixed convection of kerosene oil-based CNT nanofluid using finite element analysis. Alex. Eng. J..

[18] Hossain R., Azad A.K., Hasan M.J., Rahman M.M. (2022). Thermophysical properties of Kerosene oil-based CNT nanofluid on unsteady mixed convection with MHD and radiative heat flux. Eng. Sci. Technol. an Int. J..

[19] Hasan M., Priam S.S., Faiaz A.N.E., Azad A.K., Rahman M.M. (2023). Influence of thermal conductivity on transient mixed convection in a vented cavity with a hollow cylinder and filled with CNT-water nanofluid. Heliyon.

[20] Babu M.N., Anandan V., Parthasarathi N.L., Yildirim C.V., Babu M.D., Das S.R. (2022). Performance analysis in turning of D3 tool steel using silver nanoplatelets as additives under MQL. J. Braz. Soc. Mech. Sci. Eng..

[21] Ganesan K., Babu M.N., Santhanakumar M., Muthukrishnan N. (2018). Experimental investigation of copper nanofluid based minimum quantity lubrication in turning of H 11 steel. J. Braz. Soc. Mech. Sci. Eng..

[22] Mohan R., Shrikhande S., Joshi V., Harish R. (2022). Numerical investigation on thermal performance of duplex nanocoolant jets in drilling of Ti-6Al-4V alloy. Appl. Sci..

[23] Sultana M.N., Dhar N.R. (2022). A critical review on the progress of MQL in machining hardened steels. Adv. Mater. Processing Technol..

[24] Joshi V., Shrikhande S., Harish R., Giridharan A., Mohan R. (2022). Computational fluid dynamics simulation on thermal performance of Al/Al_2_O_3_/MWCNT nanocoolants for turning operations. Nanomaterials.

[25] Sultana M.N., Dhar N.R. (2023). Performance evaluation of high-pressure cooling by using external rotary liquid applicator in milling Ti–6Al–4V alloy. Heliyon.

[26] Mathieu G., Frédéric V., Vincent R., Eric F. (2015). 3D stationary simulation of a turning operation with an Eulerian approach. Appl. Therm. Eng..

[27] Abdulhameed A., Halim M.M., Kamil W.M.W.A., Zheng K.O., Ahmad A.U., Alsaee S.K. (2023). Influence of carbon nanotube suspensions on the structural, optical, and electrical properties of grown ZnO nanorods. Appl. Phys. A.

[28] Abdulhameed A., Halin I.A., Mohtar M.N., Hamidon M.N. (2020). The role of medium on the assembly of carbon nanotube by dielectrophoresis. J. Dispersion Sci. Technol..

[29] Abdulhameed A., Wahab N.Z.A., Mohtar M.N., Hamidon M.N., Shafie S., Halin I.A. (2021). Methods and applications of electrical conductivity enhancement of materials using carbon nanotubes. J. Electron. Mater..

[30] Pal A., Chatha S.S., Sidhu H.S. (2021). Performance evaluation of the minimum quantity lubrication with Al₂O₃- mixed vegetable-oil-based cutting fluid in drilling of AISI 321 stainless steel. J. Manuf. Process..

[31] Das A., Pradhan O., Patel S.K., Das S.R., Biswal B.B. (2019). Performance appraisal of various nanofluids during hard machining of AISI 4340 steel. J. Manuf. Process..

[32] Singh H., Sharma V.S., Singh S., Dogra M. (2019). Nanofluids assisted environmental friendly lubricating strategies for the surface grinding of titanium alloy: Ti6Al4V-ELI. J. Manuf. Process..

[33] Jerold B.D., Kumar M.P. (2011). Experimental investigation of turning AISI 1045 steel using cryogenic carbon dioxide as the cutting fluid. J. Manuf. Process..

[34] Fang Z., Obikawa T. (2020). Influence of cutting fluid flow on tool wear in high-pressure coolant turning using a novel internally cooled insert. J. Manuf. Process..

[35] Gariani S., Shyha I., Inam F., Huo D. (2017). Evaluation of a novel controlled cutting fluid impinging supply system when machining titanium alloys. Appl. Sci..

[36] Srikant R., Prasad M., Amrita M., Sitaramaraju A., Krishna P.V. (2013). Nanofluids as a potential solution for minimum quantity lubrication: a review. Proc. Inst. Mech. Eng. Part B: J. Eng. Manuf..

[37] Srikant R., Ramana V. (2015). Performance evaluation of vegetable emulsifier based green cutting fluid in turning of American Iron and Steel Institute (AISI) 1040 steel – an initiative towards sustainable manufacturing. J. Clean. Prod..

[38] Sharma V.S., Dogra M., Suri N. (2009). Cooling techniques for improved productivity in turning, international journal of machine tools and manufacture. Int. J. Mach. Tool Manufact..

[39] Luchesi V.M., Coelho R.T. (2012). Experimental investigations of heat transfer coefficients of cutting fluids in metal cutting processes: analysis of workpiece phenomena in a given case study. Proc. Inst. Mech. Eng. Part B: J. Eng. Manuf..

[40] Ahmed S.E., Elshehabey H.M. (2018). Buoyancy-driven flow of nanofluids in an inclined enclosure containing an adiabatic obstacle with heat generation/absorption: effects of periodic thermal conditions. Int. J. Heat Mass Tran..

[41] Keblinski P., R Phillpot S., S Choi S.U., Eastman J.A. (2002). Mechanisms of heat flow in suspensions of nano-sized particles (nanofluids). Int. J. Heat Mass Tran..

[42] Chummar A., Harish R. (2022). CFD simulation of laminar free convection flows of nanofluids in a cubical enclosure. Mater. Today Proc..

[43] Chummar A., Harish R. (2022). Numerical investigation of forced convective heat transfer of nanofluids within an enclosure. Mater. Today Proc..

[44] Murshed S.M.S., Leong K., Yang C. (2008). Investigations of thermal conductivity and viscosity of nanofluids. Int. J. Therm. Sci..

[45] Philip J., Shima P. (2012). Thermal properties of nanofluids. Adv. Colloid Interface Sci..

[46] Lee S., Choi S.U.S., Li S., Eastman J. (1999). Measuring thermal conductivity of fluids containing oxide nanoparticles. J. Heat Tran..

[47] Bahmani M.H., Sheikhzadeh G., Zarringhalam M., Akbari O.A., Alrashed A.A., Shabani G.A.S., Goodarzi M. (2018). Investigation of turbulent heat transfer and nanofluid flow in a double pipe heat exchanger. Adv. Powder Technol..

[48] Harish R., Sivakumar R. (2021). Turbulent thermal convection of nanofluids in cubical enclosure using two-phase mixture model. Int. J. Mech. Sci..

[49] Harish R., Sivakumar R. (2021). Effects of nanoparticle dispersion on turbulent mixed convection flows in cubical enclosure considering Brownian motion and thermophoresis. Powder Technol..

[50] Abdulhameed A., Mohtar M.N., Hamidon M.N., Mansor I., Halin I.A. (2021). Characterization and selective deposition of carbon nanotubes from carbon nanoparticles mixture using mild acid treatment and electrokinetic manipulation. Mater. Res. Express.

[51] Abdulhameed A., Halim M.M., Halin I.A. (2023). Dielectrophoretic alignment of carbon nanotubes: theory, applications, and future. Nanotechnology.

[52] Dinarvand S., Rostami M.N. (2020). Three-dimensional squeezed flow of aqueous magnetite–graphene oxide hybrid nanofluid: a novel hybridity model with analysis of shape factor effects. Proc. IME E J. Process Mech. Eng..

[53] Izady M., Dinarvand S., Pop I., Chamkha A.J. (2021). Flow of aqueous Fe2O3–CuO hybrid nanofluid over a permeable stretching/shrinking wedge: a development on Falkner–Skan problem. Chin. J. Phys..

[54] Zhu Z., He B., Chen J. (2020). Evaluation of tool temperature distribution in MQL drilling of aluminum 2024-T351. J. Manuf. Process..

[55] Khan I., Hussanan A., Saqib M., Shafie S. (2019). Convective heat transfer in drilling nanofluid with clay nanoparticles: applications in water cleaning process. BioNanoScience.

